# Transformation sarcomateuse de la maladie de Recklinghausen

**DOI:** 10.11604/pamj.2021.38.256.16742

**Published:** 2021-03-11

**Authors:** Younes Barbach, Fatima Zahra Mernissi

**Affiliations:** 1 Service de Dermatologie et Vénérologie, Hôpital Universitaire Hassan II, Fès, Maroc

**Keywords:** maladie de Von Recklinghausen, neurofibrome plexiforme, géant, neurofibromatose type 1, Von Recklinghausen disease, plexiform neurofibroma, giant, neurofibromatosis type 1

## Abstract

Neurofibromatosis type I is a common genetic disease. Affected patients are 4 times more likely to develop a tumor. Most tumors are benign (neurofibromas). Although these rarely result in malignant tumors, they represent the leading cause of death in patients, thus making neurofibromatosis type I a severe disease. It mainly develops to melanoma, pheochromocytoma, astrocytoma, optic glioma, Wilms tumor of and leukemia. Sarcomatous transformation is exceptional. Early diagnosis is essential. It arises from isolated nodular or plexiform neurofibromas. During the monitoring of patients with NF1 and with plexiform neurofibromas, clinicians should consider the possibility of its transformation into neurofibrosarcoma. This is also the case for rapid increase in tumor size, its hardening, its extremely painful nature or the occurrence of neurological signs. We report the case of a 42-year-old female patient, with childhood history of Von Recklinghausen disease, presenting with pain, bleeding and an increase in plexiform neurofibroma size in the right lower limb (A, B, C). Clinical examination showed coffee-with-milk colored spots, cutaneous neurofibromas and large size ulcerated and painful mass involving all the right lower limb. Computed tomography (CT) scan of the limb objectified a tumoral process at the level of the posterior region of the lower limb. Biopsy of the mass showed malignant peripheral nerve sheath tumor.

## Image en médecine

La neurofibromatose type I est une maladie génétique fréquente. Les patients qui en sont atteints ont 4 fois plus de risque de développer une pathologie tumorale. La majorité des tumeurs sont bénignes (neurofibromes), même si les complications tumorales malignes sont rares, ceux sont-elles qui font toute la gravité de la NF1 car elles représentent la principale cause de décès des patients atteints. Il s´agit surtout de la transformation en mélanome, phéochromocytome, astrocytome, gliome des voies optiques, tumeur de Wilm et leucémie. La transformation sarcomateuse est exceptionnelle. Le diagnostic doit être précoce, il se développe à partir de neurofibromes nodulaires isolés ou plexiformes. La surveillance des patients atteints de NF1 et porteurs de neurofibromes plexiformes doit faire suspecter une transformation en neurofibrosarcome. Il est de même que l´augmentation rapide de la taille de la tumeur, le durcissement de sa consistance, le caractère très douloureux ou l´apparition de signes neurologiques. Nous rapportons le cas d´une patiente âgée de 42 ans, atteinte d´une maladie de Von Recklinghausen depuis son enfance, ayant consulté pour l´installation d´une douleur, un saignement et une augmentation de taille d´un neurofibrome plexiforme du membre inférieur droit (A, B, C). L´examen clinique objectivait la présence de tâches café au lait, des neurofibromes cutanés et une masse de grande taille prenant tout le membre inférieur droit ulcérée et douloureuse. Le scanner du membre montrait la présence d´un processus tumoral au niveau de la région postérieure du membre inférieur. La biopsie de la masse concluait à une tumeur maligne de la gaine nerveuse périphérique.

**Figure 1:**
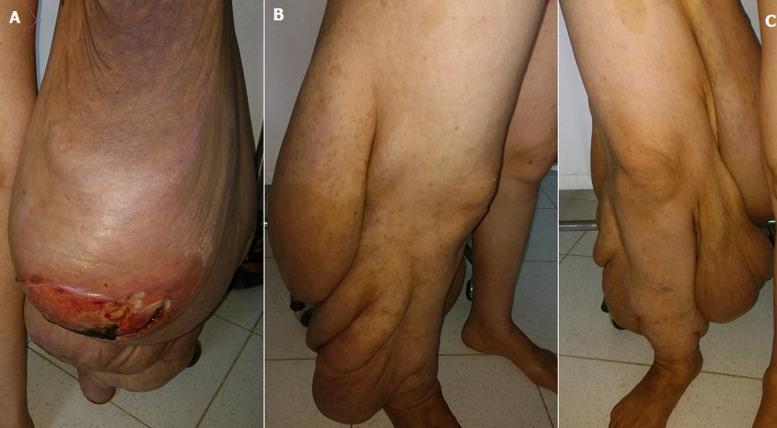
tumeur prenant tout le membre inférieur droit ulcérée et nécrotique par endroit (A, B, C)

